# A FFAR1 full agonist restores islet function in models of impaired glucose-stimulated insulin secretion and diabetic non-human primates

**DOI:** 10.3389/fendo.2022.1061688

**Published:** 2022-11-22

**Authors:** Brian Rady, Jianying Liu, Hui Huang, Ivona Bakaj, Jenson Qi, S. P. Lee, Tonya Martin, Lisa Norquay, Mark Player, Alessandro Pocai

**Affiliations:** ^1^ Cardiovascular and Metabolism Discovery, Janssen Research and Development, Spring House, PA, United States; ^2^ Discovery Chemistry, Janssen R&D, Spring House, PA, United States; ^3^ Medical Affairs, Janssen R&D, Spring House, PA, United States; ^4^ Business Development, Janssen R&D, Raritan, NJ, United States

**Keywords:** Diabetes, human islet, insulin, glucagon, GLP-1, non-human primate, NASH - non-alcoholic steatohepatitis, CKD - chronic kidney disease

## Abstract

The free fatty acid receptor 1 (FFAR1/GPR40) mediates fatty acid-induced insulin secretion from pancreatic β-cells. At least 3 distinct binding sites exist on the FFAR1 receptor and numerous synthetic ligands have been investigated for their anti-diabetic actions. Fasiglifam, binds to site-1 and stimulates intra-cellular calcium release and improves glycemic control in diabetic patients. Recently, small molecule FFAR1 agonists were discovered which bind to site-3, stimulating both intra-cellular calcium and cAMP, resulting in insulin and glucagon-like peptide-1 (GLP-1) secretion. The ability of our site-3 FFAR1 agonist (compound A) to control blood glucose was evaluated in spontaneously diabetic cynomolgus monkeys during an oral glucose tolerance test. In type-2 diabetic (T2D) animals, significant reductions in blood glucose and insulin were noted. To better understand the mechanism of these *in vivo* findings, we evaluated the effect of compound A in islets under several conditions of dysfunction. First, healthy human and non-human primate islets were treated with compound A and showed potentiation of insulin and glucagon secretion from both species. Next, we determined glucose-responsive insulin secretion under gluco-lipotoxic conditions and from islets isolated from type-2 diabetic humans. Despite a dysfunctional phenotype that failed to secrete insulin in response to glucose, site-3 FFAR1 agonism not only enhanced insulin secretion, but restored glucose responsiveness across a range of glucose concentrations. Lastly, we treated *ex vivo* human islets chronically with a sulfonylurea to induce secondary beta-cell failure. Again, this model showed reduced glucose-responsive insulin secretion that was restored and potentiated by site-3 FFAR1 agonism. Together these data suggest a mechanism for FFAR1 where agonists have direct effects on islet hormone secretion that can overcome a dysfunctional T2D phenotype. These unique characteristics of FFAR1 site-3 agonists make them an appealing potential therapy to treat type-2 diabetes.

## Introduction

Free Fatty Acid Receptor 1 (FFAR1/GPR40) is a GPCR whose endogenous ligands are medium to long chain free fatty acids (FFAs) (1). It is highly expressed in pancreatic beta-cells and to a lesser degree in enteroendocrine K and L cells, where it mediates FFA-induced secretion of insulin and incretins respectively (2, 3). Two types of synthetic FFAR1 agonists have been developed, the first are partial agonists which bind to site-1 and do not maximally mobilize intracellular calcium nor trigger cAMP (4). The second are GPR40 full agonist which increase both Ca^2+^ (to a greater degree than partial agonists) and cAMP (4). Due to this differential receptor pharmacology, site-3 FFAR1 agonists have been suggested to have the potential for enhanced efficacy vs partial agonists. Several small molecule GPR40 agonists have been investigated for their anti-diabetic effects (5, 6). TAK-875 (fasiglifam, Takeda) was a clinically effective GPR40 partial agonist used in two Phase II dose-ranging studies – one in Central/North America and another in Japan. These studies showed reductions in HbA1c from baseline of 0.83% in the Japanese group and 1.4% in Central/North America after 12 weeks (7, 8). Despite this efficacy, Fasiglifam was ultimately withdrawn from a Phase III clinical trial due to drug-induced liver injury (9). However, the glucose dependency of GPR40-mediated effects on insulin secretion together with the combinability with other diabetic medications provides an attractive therapeutic opportunity for the treatment of diabetes.

Since the failure of TAK-875 in humans a number of synthetic allosteric full agonists for GPR40 have been identified. These full agonists have been described to bind to distinct sites compared to partial agonists and the endogenous fatty acid ligands (10). With multiple binding sites identified, this suggests a significant degree of flexibility in the active confirmation of GPR40 and the potential for differential activity depending on the conformation achieved (11). Indeed, cooperativity between orthosteric and allosteric site binders has been demonstrated for GPR40 (12). This raises the possibility that novel synthetic full agonists may achieve unique pharmacology and therapeutic benefit based on their differential conformation and signaling.

Along with insulin resistance, β-cell failure is central to the onset and development of type 2 diabetes (T2D), most likely due to a concert of genetic, epigenetic, and environmental factors that collectively impact β-cell survival and function (13). Functional studies performed with isolated islets and in humans have consistently reported a marked decrease of insulin release from T2D β-cells, particularly in response to glucose stimulation (14–16). These data support the emerging concept that in human T2D β-cells may have developed a dysfunctional phenotype characterized by a lack of insulin staining and inability to secrete insulin under physiological conditions (17). The theoretical reversal of this phenotype could lead to improved treatment or slowing of progression in type-2 diabetics. Indeed, human T2D β-cells show improved insulin secretory function following exposure to metformin, with partial recovery of insulin release in response to glucose stimulation and replenishment of insulin granules – highlighting the capacity of diseased islets to recover (14). Together, these data point to the potential of therapeutics directed at β-cell decline as a mechanism by which to intervene in the pathophysiology of T2D.

In this study, we describe the characterization of compound A, a newly identified site-3 FFAR1 agonist. Compound A demonstrated anti-diabetic effects in non-human primates and ZDF rats. The mechanisms of these beneficial metabolic effects are explored in healthy NHP and human islets, as well as several models of islet dysfunction including primary T2D human islet, gluco-lipotoxic islets, and islets displaying sulphonylurea secondary failure.

## Methods

### Cell lines and IP-One assays

The hFFAR1 low-expressing stable CHO-K1 cell line used in this study was purchased from Multispan, Inc (Cat # C1101-1A). Cells were maintained in DMEM/F-12 supplemented with 10% FBS, 1% 10000 U/mL penicillin and 100 μg/mL streptomycinand 10 μg/ml puromycin and incubated at 37°C with 5% CO2. The day before the assay, hFFAR1-expressing CHO-K1 cells were plated overnight in 384-well plates (4,000 cells per well) in complete media, with or without 100 ng/mL Pertussis Toxin (Tocris, Cat # 3097). The following day, the culture media was replaced with assay buffer containing HBSS with calcium and magnesium, 20 mM HEPES and 0.1% Fatty acid free BSA, pH 7.4. Compounds were then added and incubated with cells at 37°C for 90 min. Analytes were detected according to the manufacturer’s protocol (CisBio Ipone Tb kit, Cat # 62IPAPEC). Data presented are representative of at least three independent experiments performed in quadruplicate for each compound. Data are represented as averages ± S.D.

### Calcium measurements

The day before the assay, hFFAR1-expressing CHO-K1 cells were plated overnight in 384-well plates (20,000 cells per well) in complete media. The following day, the culture media was replaced with 25 μL of assay buffer containing HBSS with calcium and magnesium, 20 mM HEPES and 0.1% Fatty acid free BSA, pH 7.4, and starved for 1 h at 37°C. Calcium-sensitive fluorescent dye (Fluo 6, Molecular Devices, Cat # R8190) was then added in 25 μL assay buffer and the cells incubated for another hour at 37°C protected from light. Plates were read on the FLIPR Tetra (Molecular Devices) measuring emission at 515-575 nm caused by excitation at 470-495 nm before and up to 8 min after addition of 12.5 μL of 5X agonist solution (prepared in assay buffer). The concentration response curves were constructed based on the maximal responses over baseline obtained for different concentrations of each compound. Data presented are representative of three independent experiments performed in quadruplicate for each compound. Data are represented as averages ± S.E.M.

### cAMP HTRF measurements

The day before the assay, hFFAR1-expressing CHO-K1 cells were plated overnight in 384-well plates (20,000 cells per well) in complete media. The following day, the culture media was replaced with assay buffer containing HBSS with calcium and magnesium, 20 mM HEPES and 0.1% Fatty acid free BSA, pH 7.4, and starved for 1 h at 37°C. The assay buffer was then replaced with fresh assay buffer containing 500 μM IBMX, and compounds were added in assay buffer (no IBMX) for 30 min. Analytes were detected according to the manufacturer’s protocol (CisBio cAMP Dynamic kit kit, Cat # 62AM4PEC). Fluorescence was read with a PHERAstar plate reader using an excitation of 337 nm and emissions of 620 and 665 nm. Raw data were converted to nM cAMP by interpolation from a cAMP standard curve. Emax and EC50 determinations were made from an agonist-response curve analyzed with a curve fitting program using a 4-parameter logistic dose response equation in Graphpad Prism 7.0. Data presented are representative of three independent experiments performed in quadruplicate for each compound. Data are represented as averages ± S.D.

### Radioligand binding experiment

Membranes were prepared as follows. Cells stably expressing hFFAR1 were harvested by centrifugation (10 min at 5,000 g). The pellet was resuspended in lysis buffer [10 mM Tris-HCl, pH 7.4, 137 mM NaCl, and Complete protease inhibitor cocktail (Roche, Cat # 11873580001: 1 tablet per 40 ml)], and lysed using 30 strokes with a Dounce homogenizer on ice. The homogenate was centrifuged at 4°C (10 min at 900 g). The supernatant was centrifuged at 4°C for 60 min at 100,000 g. The resulting pellet was resuspended in wash buffer [10 mM Tris-HCl, pH 7.4, 1 M NaCl, and Complete protease inhibitor cocktail (1 tablet per 40 ml)]. The homogenate was centrifuged at 4°C for 30 min at 100,000 g. Membranes were resuspended at 10 mg/ml protein in 10 mM Tris-HCl, pH 7.4, and 137 mM NaCl.

Test compounds were serially diluted in binding buffer (PBS + 0.1% fatty acid-free BSA). Each well of the 96-well assay plate contained diluted test compounds, 50 nM [3H]-Compound A or 10 nM [3H]-AM-1638, and 10 μg/well hFFAR1 membrane suspension in a total volume of 100 μl. The binding reaction was allowed to equilibrate for 60 minutes at room temperature with shaking. Binding assays were terminated using a Harvester Filtermate 96 (PerkinElmer). Bound and free radioligands were separated by collecting the membrane-bound fraction onto GF/B filterplates impregnated with PEI 0.5% and pre-wetted with binding buffer. Filterplates were washed 4 times with ice-cold binding buffer and dried for 2 hours. Microscint O (50 µL) was added to each well and radioactivity was counted using the Topcount (PerkinElmer). Nonspecific binding was determined using 10 μM cold Compound A or AM-1638. Data analysis was performed using GraphPad Prism 7.0 (GraphPad Software, Inc., La Jolla, CA, 92037, USA). Data presented are representative of three independent experiments performed in triplicate for each compound. Data are represented as averages ± S.D.

### Hormone secretion in islets

Cynomolgus monkey islets were obtained from AX Biotech. Human islets were obtained from AX Biotech, Prodo Labs, and the University of Illinois at Chicago and cultured in CMRL Media (Thermo Fisher Scientific, Cat # 11530-037), 10mM Niacinamide, [1 mg/ml, 0.55 mg/ml, 0.67 ug/ml] ITS (Thermo Fisher Scientific, Cat # 41400045); 16.7 mM Zinc Sulfate; 5 mM Sodium Pyruvate; 2 mM Glutamax (Thermo Fisher Scientific, Cat # 35050-061); 25 mM HEPES; 10% FBS. For hormone secretion assays, whole islets were dispersed with Accutase (Thermo Fisher Scientific, Cat # A1110501) for 10 minutes at 37°C. 20,000 cells per well were plated in V-bottom 96-well plates and cultured overnight in complete medium. In the case of gluco-lipo toxic (GLT) treated cells, complete media was supplemented with glucose up to 11mM and 2:1 oleate (Sigma O7501-1G) to palmitate (SigmaP9767) at final concentration of 0.5mM. GLT treated cells were cultured for 48h, normal culture islets were assayed the following day. The day of assay, medium was replaced with assay buffer (Krebs Ringer) and cells were pre-incubated in 2 mM glucose for 1 hour. Next, the indicated concentrations of compounds were added in either 2 mM or 12 mM glucose and the cells were incubated at 37°C for 1 hour. The supernatant was then collected and tested for insulin or glucagon using the CisBio HTRF Insulin or Glucagon assay kit (Cat # 62INSPEC and 62CGLPEH). Data are represented as averages ± S.E.M. from 3 different islet donors. Statistical significance was determined by one-way ANOVA with Dunnett *post hoc* analysis using GraphPad Prism 7.0 (GraphPad Software, Inc., La Jolla, CA, 92037, USA).

### Rodent studies

Male FFAR1 knockout mice were obtained from Taconic and housed singly with ad libitum access to food and water on a 12‐h light/dark cycle. 6 hours before the oral glucose tolerance test (oGTT), the mice were transferred to clean cages and fasted. For the oGTT, the mice were weighed and randomized into groups based on fasted blood glucose and body weight. Mice were dosed with vehicle (0.5% methocel) or compounds thirty min prior to the oGTT (glucose, 2 g/kg, po). Blood was collected from the tail vein at 0, 10, 30, 60 and 120 min after glucose challenge to measure blood glucose; plasma was used to determine insulin and glucagon levels (Meso Scale Discovery). The area under the curve for blood glucose excursion was calculated from t = 0 to t = 120 minutes. Percent lowering of glucose was calculated from the AUC data with respect to the vehicle-treated group.

Male ZDF rats (200–250 g) were housed 2 per cage in a temperature-controlled room with a 12-hour light/dark cycle. They were allowed ad libitum access to water and fed with normal rodent chow. The night before the oral glucose tolerance test (oGTT), the rats were transferred to clean cages and fasted overnight. On the morning of the oGTT, the rats were weighed and randomized into groups based on fasted blood glucose and body weight. Rats were dosed with vehicle (0.5% methocel) or compounds thirty min prior to the oGTT (glucose, 2 g/kg, po). Blood was collected from the tail vein at 0, 10, 30, 60 and 120 min after glucose challenge to measure blood glucose; plasma was used to determine insulin and glucagon levels (Meso Scale Discovery). The area under the curve for blood glucose excursion was calculated from t = 0 to t = 120 minutes. Percent lowering of glucose was calculated from the AUC data with respect to the vehicle-treated group. All *in vivo* experiments were performed with approval from the IACUC.

### Non-human primate study

Cynomolgus monkeys within a larger colony were determined to be diabetic using a combination of age, body weight, serum glucose, insulin, HbA1c, TC, TG, LDLc, HDLc, ALT, AST, BUN, and creatinine. For OGTT, animals were fasted overnight and then orally gavaged with TAK-975 or compound A 60 minutes prior to glucose administration. At time = 0, animals were orally gavaged with glucose (1.75 g/kg) with blood samples taken pre-glucose bolus, and 15, 30, 60, 120 minutes following. Plasma GLP1 and insulin was assessed (Meso Scale Discovery) from blood draws at all time points (insulin) or the -60 minute pre-dose timepoint and the 120 minute post-glucose timepoints (GLP1). All NHP studies were IACUC approved.

## Results

### Compound A is potent and specific FFAR1 agonist

**Figure 1:**
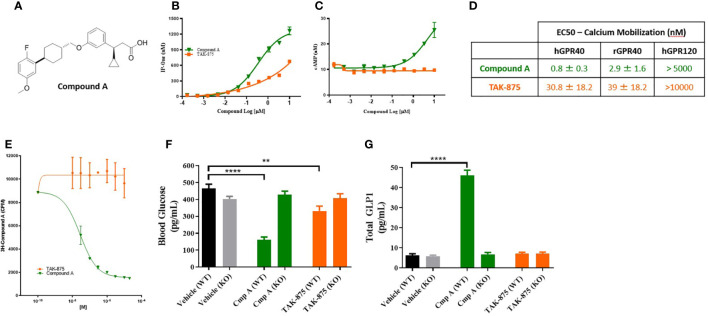
Structure and characterization of Compound A. Structure of compound A **(A)**. Inositol monophosphate and cAMP measurements in GPR40 receptor overexpression CHO cells **(B, C)**. Summary table of calcium mobilization EC_50_s in overexpressing HEK cells **(D)**. **(E)** Competition binding assay where cells are pre-treated with 3H-Compound A then the ability of either a site 1 (TAK-875) or site 3 (cmp A) to compete off hot compound A is assessed. **(F, G)** Effects of compound A and TAK-875 on blood glucose levels during OGTT and GLP1 secretion in wild-type (WT) and GPR40 knockout mice (KO). Mice were dosed at 100 mg/kg for both compounds. **p<0.01 ****p<.0001 by 1-way ANOVA vs Vehicle (WT).

A novel synthetic selective FFAR1 full agonist, compound A, was characterized through *in vivo* and *in vitro* pharmacology studies (18) (**Figure 1A**). In a calcium mobilization assay conducted in a CHO cell line expressing human FFAR1 receptor compound A and the FFAR1 partial agonist (TAK875) elicited a dose-dependent increases in inositol monophosphate (IP1), a stable metabolite of IP3 and indicator of calcium mobilization. Compound A achieved a higher E_max_ confirming the full agonist nature of the compound (**Figure 1B**). We also demonstrated activation of G_s_ signaling by compound A but not by the partial agonist consistent with the site-3 binding ability of compound A (4) (**Figure 1C**). To directly confirm the different binding site of compound A we performed competition studies with a tritiated version of the small molecule. Following incubation with 3^H^-compound A, partial agonists showed no ability to compete off the 3^H^-compound A while cold compound A and a known FFAR1 full agonist, AMG-1638 (19), were both able to compete off radiolabeled compound A (**Figure 1E**). In Ca^2+^ mobilization assays in high expressing HEK cells, compound A was active at the human and rat FFAR1 receptors with EC_50_s of 800pM and 2.9nM respectively (**Figure 1D**). Additionally, we demonstrated that compound A displayed specificity for FFAR1 versus related long-chain fatty acid receptors named GPR120, GPR43, and GPR41. We then performed a counter-screen of compound A against activity for 50 other GPCRs and 40 enzymes and ion channels, which showed no meaningful functional activity at concentrations up to 10µM (data not shown). Finally, to ensure the specificity of this compound for FFAR1, wild-type and FFAR1 knockout mice were dosed with compound A, TAK-875, or vehicle. In contrast to wild-type mice which showed glucose lowering and potentiation of glucagon-like peptide-1 (GLP-1) secretion, the effect of compound A and the partial agonist on glucose lowering or glp-1 secretion were abolished in *ffar1*-/- mice (**Figures 1F, G**).

### Site-3 FFAR1 agonism improves glucose metabolism in cynomolgus monkeys

**Figure 2:**
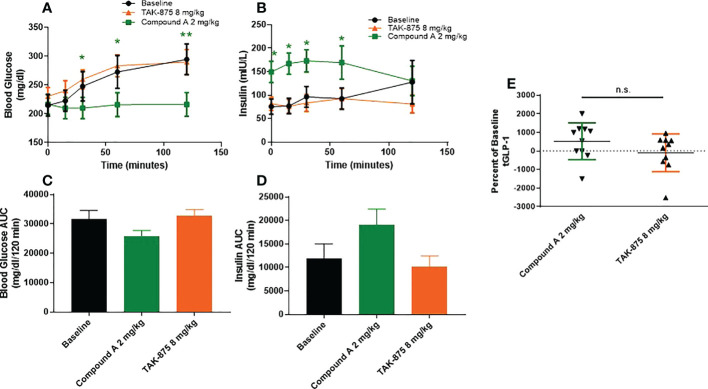
Glucose metabolism and GLP1 secretion in spontaneous T2D cynomolgous monkeys. Oral glucose tolerance test was performed in diabetic non-human primates with compound A.Compound was dosed 60 minutes prior to challenge with glucose (at 0 minutes). **(A)** Plasma glucose, **(B)** insulin secretion, and **(C)** plasma total GLP1 were measured. **(D, E)** AUCs of blood glucose and insulin secretion were caluclated from x=0, y=0 of respective graphs. n=10 per group, * = p ≤ 0.05, ** = p ≤ 0.01, n.s. = p>0.05 compound A to baseline by t-test.

To understand how the metabolic effects of site-3 agonism differed from site-1 agonism, we evaluated the effects of acute dosing in both spontaneously type-2 diabetic and lean cynomolgus monkeys. In T2D monkeys, fasting blood glucose levels were above 200mg/dl on average for all animals and these NHPs showed little or no insulin secretion following glucose challenge confirming their diabetic phenotype (**Figure 2A**). These animals were treated with a site-1 or our site-3 FFAR1 agonist 60 minutes prior to performing an OGTT. Treatment with compound A attenuated blood glucose excursion 120-minutes following glucose administration (294.6 vs 216.4 mg/dl; vehicle vs. Cmp A; p<0.01) while enhancing insulin secretion (76.7 vs. 167.3 mIU/L; vehicle vs Cmp A; p<0.05; **Figures 2A–D**). Increased levels of insulin were also noted at time zero before glucose administration, but 60 minutes after site-3 FFAR1 compound administration (75.6 vs. 149.5 mIU/L; vehicle vs. Cmp A; p<0.05) (**Figure 2B**). Interestingly, this did not translate into hypoglycemia in these animals (214.9 vs. 216.4 mg/dl; vehicle vs Cmp A; p=0.799; **Figure 2A**). At no point during the OGTT did insulin levels rise above baseline in the untreated group demonstrating the profound islet dysfunction in these diabetic animals (**Figure 2B**). In contrast, treatment with a compound A agonist did not alter glucose clearance or insulin secretion (294.6 vs 290.1 mg/dl; vehicle vs. Cmp A; p=0.89 and 127.9 vs 81.8 mIU/L; vehicle vs. Cmp A; p=0.15 respectively). Additionally, we saw a numerical elevation of 5.3-fold in plasma GLP-1 over baseline (**Figure 2E**).

### Characterization of site-3 FFAR1 agonists in monkey and human islets

**Figure 3:**
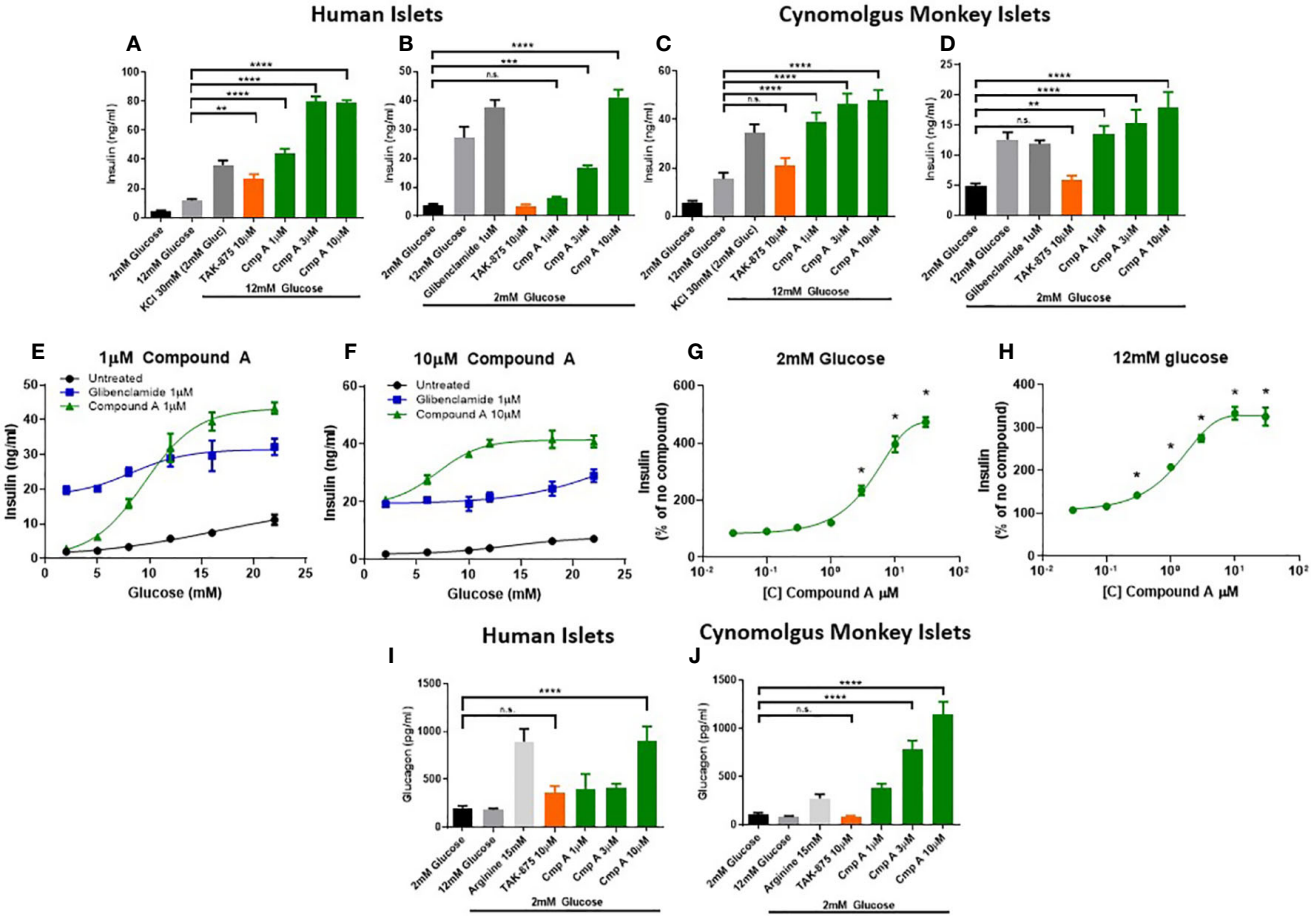
*Ex vivo* islet insulin and glucagon secretion. Insulin secretion from human islets was stimulated with 12mM **(A)** and 2mM **(B)** glucose. Cynomolgus monkey islets were also assessed for glucose stimulated insulin secretion in 12mM **(C)** and 2mM glucose **(D)**. At low (1μM) and high (10μM) concentrations of compound A, glucose dose response curves from a single healthy human islet donor were measured in human islets showing a differential potentiation of insulin secretion at lower glucose levels. Compound dose response curves at 2mM **(G)** and 12mM **(H)** glucose expanded on this finding to show the effects in low glucose were apparent above 3µM only. Glucagon secretion in 2mM glucose from healthy human **(I)** and cynomolgus **(J)** islets. n=3 for all, except **(E, F)** where n=1. **p<0.01 ***p<.001 ****p<.0001 by 1-way ANOVA vs 2mM or 12mM glucose as indicated in **(A–D)** & **(I, J)**. * = p ≤ 0.05 by 2-way ANOVA for **(G, H)**. n.s., p>0.05.

To better understand the mechanism and translatability of these *in vivo* findings, we evaluated the effects of site-3 FFAR1 agonism in islets under several conditions of dysfunction. First, healthy human (**Figures 3A, B**) and non-human primate islets (**Figures 3C, D**) were treated with compound A and glucose-responsive insulin secretion assays were performed. These were conducted in isolated islets obtained from non-diabetic cynomologus monkey and human donors whose insulin secretion was responsive to glucose and the known secretagogue potassium chloride (KCl). In human islets, treatment with Compound A increased insulin secretion in a dose responsive manner in the presence of 12mM glucose (**Figure 3A**). Similar to cynomolgus islets, compound A at 3 and 10μM stimulated insulin secretion in the presence of low glucose achieving a maximal response of 10.6-fold increase at 10μM (3.93 vs. 41.15 ng/ml; p<0.0001; **Figure 3B**) while a site-1 FFAR1 agonist did not result in increased insulin levels. In monkey islets, site-3 FFAR1 agonism maximally stimulated insulin secretion by 3.1-fold vs vehicle (15.5 vs. 47.7ng/ml; p<0.0001) in the presence of high glucose (12mM) (**Figure 3C**). We also noted insulin secretion in the presence of low (2mM) glucose by 3.7-fold vs vehicle (4.8 vs 17.86 ng/ml; p<0.0001; **Figure 3D**) suggesting both a glucose-dependent and glucose-independent mechanism of insulin secretion. Non-glucose responsive mechanisms of insulin secretion, such as sulphonylureas, have been linked to the potential for hypoglycemia in humans. Therefore we sought to better characterize the glucose-responsiveness of site3 FFAR1 agonism. To this end, we created glucose dose-response curves at fixed concentrations of our compound (1 and 10 μM) to understand the glucose threshold above which this mechanism potentiates insulin secretion *in vitro*. Compound A at 1μM stimulated insulin secretion in high glucose but not in low glucose conditions (**Figure 3E**). At 10µM, compound A potentiated insulin secretion in low glucose (**Figure 3F**) however, unlike the sulphonylurea glibenclamide, compound A still showed a glucose-dependent enhancement of insulin secretion in higher concentrations of glucose (**Figure 3F**). Next, compound dose-response curves were constructed to determine the minimal efficacious concentration in fixed glucose concentrations (2mM and 12mM). The 2mM glucose curve showed potentiation of insulin secretion at concentrations of compound above 3µM, while the 12mM glucose curve showed potentiation at lower compound concentrations (> 0.3µM) (**Figures 3G, H**) suggesting a ~10-fold window in compound concentration that potentiates insulin secretion in high but not low glucose.

To expand on the finding of increased circulating glucagon levels in mice (**Figure 1G**), we measured glucagon secretion from NHP and human islets. FFAR1 receptor is expressed in α-cells and endogenous fatty acids ligands have been shown to induce glucagon secretion in rodent islets and in healthy adults (20–22). Incubation with the positive control arginine demonstrated that islets from both species had the capacity to secrete glucagon (**Figures 3I, J**). Site-3 FFAR1 treatment in human and monkey islets, increased glucagon secretion in the presence of 2mM glucose by 4.5- and 10.9-fold respectively (202.4 vs. 902.1 pg/ml; p<0.0001 and 104.72 vs. 1145.23 pg/ml; p<0.0001 respectively). This effect was specific to the site-3 FFAR1 agonist, as the site-1 FFAR1(TAK-875) showed no impact on glucagon secretion in islets isolated from either species (**Figures 3I, J**).

### FFAR1 site-3 agonism restores insulin secretion in type-2 diabetic and gluco-lipotoxic islets

**Figure 4:**
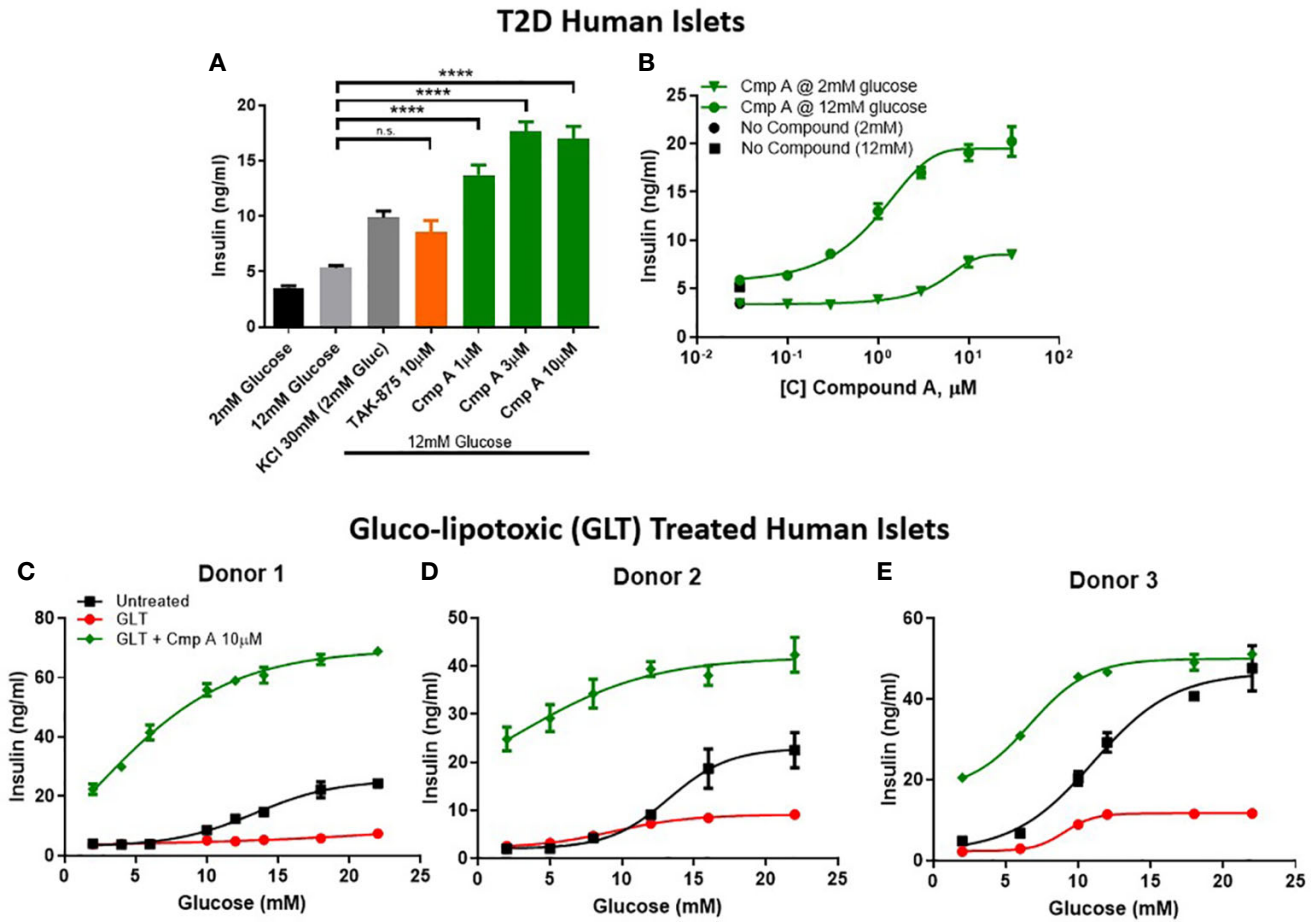
Insulin secretion from dysfunctional human islets. Diseased islets isolated from T2D donors **(A)** challenged in the presence of compounds at 12mM glucose (n=3). **(B)** Within one T2D donor, compound dose response curves in 12mM and 2mM glucose were measured. **(C–E)** To mimic β-cell dysfunction obsevered in T2D islets, healthy human islets were exposed to 48 hours of high glucose and lipid concentrations (gluco-lipo toxic GLT).-3 human donors were treated under GLT conditions then glucose responsive insulin secretion was measured in the presence or absence of compounds. **** p<0.0001 by 1-way ANOVA, n.s., p>0.05.

To further explore the therapeutic potential of our site-3 FFAR1 agonist in pathological conditions, we tested compound A in human islets obtained from type-2 diabetic donors. First, we determined the glucose-responsive insulin secretion of these diabetic islets. We confirmed that T2D islets are dysfunctional and have a reduced glucose-stimulated insulin secretion with only a ~1.5-fold increase in insulin secretion when treated with high glucose. Under these conditions, compound A not only potentiated insulin secretion (5.3 vs 16.97 ng/ml; p<0.0001) (**Figure 4A**), but also restored glucose responsiveness across a range of increasing glucose concentrations (**Figure 4B**). To better understand the relationship between compound concentration, glucose levels, and insulin release we created a compound dose-response curve for diabetic islets in 2mM and 12mM glucose. In these islets, the potentiation of insulin in 12mM was similar to what was seen in healthy human islets, but secretion in 2mM glucose was dramatically reduced (474% vs 165%; healthy vs. T2D; **Figures 3G**, **4B**). This suggests, that with disease-state islets the potential for insulin secretion in low glucose may be diminished.

Due to relative scarcity of human T2D islets, we sought to implement an *ex vivo* model of islet dysfunction that mirrored the GSIS defects that are characteristic of diabetic islets. To this end, we cultured non-diabetic human islets in hyperglycemic (11mM) and hyperlipidemic (0.5mM 2:1 oleate:palmitate) conditions. The resulting gluco-lipo toxicity (GLT) led to an average 2.5-fold reduction of GSIS AUC across 3 human donors (**Figures 4C–E**). Similar to what was observed in healthy and diabetic islets, site-3 FFAR1 agonism potentiated insulin secretion at all glucose levels. However, unlike glibenclamide which showed no glucose dependency in potentiating insulin section, FFAR1 site-3 agonism further increased insulin secretion as glucose concentrations increased (**Figures 4C–E**).

### Site-3 FFAR1 agonists potentiate insulin secretion following sulphonylurea-induced islet dysfunction

**Figure 5:**
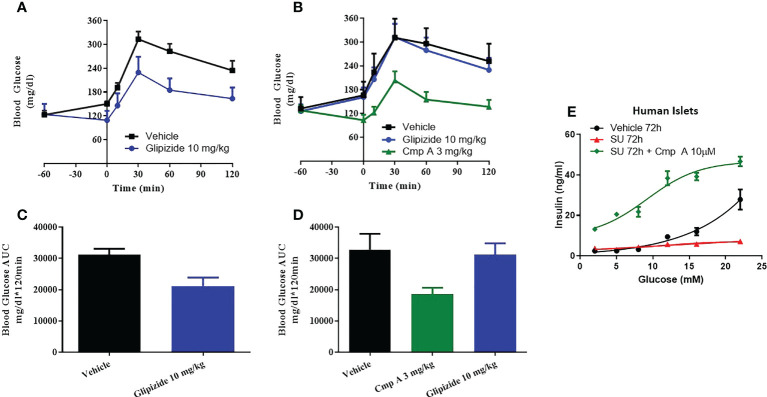
Sulfonylurea-induced secondary beta-cell failure. ZDF rats were chronically treated with vehicle **(A)** or the sulfonylurea (SU) Glipizide **(B)** for 25 days. On day 26 an OGTT was performed following acute treatment with Glipizide or compound **(A)** Quantification of the AUC for the OGTT after 25 day vehicle **(C)** or Glibenclamnide **(D)** treatment was measured. *Ex vivo* human islets were treated with another sulfonylurea, Glibenclamide, for 72h then challenged with acute glibenclamide treatment or Compound A **(E)**.

In the last model of dysfunction tested, we sought to build upon a finding by Tsuda et al. who previously reported that a FFAR1 agonist enhanced glucose tolerance in sulfonylurea-desensitized normal and diabetic rats (23). While Tsuda showed that a GPR40 agonist was able to prevent the loss of glucose control associated with sulfonylurea (SU) secondary failure, here we expand on this finding by first establishing secondary failure before initiating treatment - akin to a treatment paradigm that would be used to treat humans with existing secondary failure. We induced SU-desensitization in obese dyslipidemic Zucker Diabetic Fatty (ZDF) rats by treating with the sulfonylurea glyburide (SU) for 4 weeks then assessed glucose tolerance by oral glucose tolerance test (OGTT). Following chronic SU treatment, acute dosing with a SU no longer reduced blood glucose levels compared to chronic vehicle-acute vehicle animals, AUC 31208 vs. 21141 mg/dl*120min; p=0.009 (**Figures 5A, B**). In this setting of SU desensitization, treatment with compound A was able to lower blood glucose AUC 32844 vs. 18513 mg/dl*120min; p=0.02 p=0.004 and (**Figures 5C, D**).

To address the translatability of these findings, we evaluated the impact of site-3 FFAR1 agonism in dysfunctional human islets following prolonged use of sulfonylureas *in vitro*. When non-diabetic human islets were incubated for 72 hours in the presence of high concentrations of glibenclamide, the islets lost responsiveness to glucose stimulation (**Figure 5E**). However, our site-3 FFAR1 compound was able to potentiate insulin secretion across a range of glucose concentrations. (**Figure 5E**).

## Discussion

FFAR1 is highly expressed in pancreatic beta-cells and in enteroendocrine K and L cells where it mediates FFA-induced secretion of insulin and incretins respectively (2, 3). Previous clinical data (12 wk) generated with the partial agonist TAK-875 (fasiglifam) demonstrated improvement in HbA1c in T2D patients and recently, SCO-267, a full agonist compound was shown to improve glycemic control after single and multiple doses by stimulating the secretion of insulin, glucagon, and gut peptides in healthy adults, without increasing the risk of hypoglycemia (7, 8, 22). Here, we assessed the pharmacology of partial and full agonism in NHP and human islets and provided evidence that compound A, a novel synthetic site-3 FFAR1 agonist results in enhanced efficacy vs site-1 agonism in diabetic NHPs. We also demonstrated for the first time that full agonism is able to restore human β-cell failure, considered central to the onset and development of type 2 diabetes (T2D) (13). As a key driver of pathophysiology, restoration and preservation of β-cell function has the potential to intercept the progression of diabetes. While the clinical translatability and the safety of our results remains to be determined, FFAR1 full agonists offer a novel approach for the intervention and restoration of β-cell function in T2D.

One mechanistic hypothesis for the effects we observed with site-3 FFAR agonism involves action on the two phases of insulin secretion. The first phase involves fusion and exocytosis of mature insulin vesicles already docked and primed to the plasma membrane. The second is a slower process by which mature insulin vesicles are moved from more distal intra-cellular locations to the plasma membrane and then secreted (24). Insulin vesicle mobilization in the second phase involves the reorganization of the intracellular cytoskeleton filaments, stress fibers, and focal adhesions (25–27). The effects FFAR1 full agonism has on secretion of islet hormones may be due to its ability to potentiate first phase secretion through Ca2+ mediated effects (similar to site-1 agonism) as well as recruitment of mature insulin granules to the plasma membrane through cAMP mediated effects (unique to site-3 agonism vs site-1). This is supported by the observation in FFAR1 knockout mice that FFA-induced second phase insulin secretion is reduced by about half, through loss of PKD phosphorylation and decreased actin remodeling (28). While in humans, morphometric studies in T2D islets have highlighted impairment of this second phase of insulin secretion as a component of islet dysfunction in T2D (29).

FFAR1 has been shown to be expressed in mouse α-cells and its agonism by FFAs increases glucagon secretion in primary mouse and rat islets, through Ca2++ dependent secretion similar to β-cells (20, 21). Importantly, α-cells treated with GPR40 agonists retain their sensitivity to glucose-dependent inhibition of glucagon secretion (20). Our own data build upon these findings to confirm that this secretory effect is present in higher species, cynomolgus monkey and human, as well. FFAR1 full agonists have been shown to reduce body weight in diabetic rats (30). Glucagon secretion may play a significant role in the therapeutic mechanism of action for FFAR1, as it contributes directly to weight loss by decreasing food intake (31–33). Additionally, glucagon secretion may help to limit the impact that non-glucose responsive insulin secretion would have on blood glucose levels. While our data suggests potentiation of insulin in low glucose was only seen at higher compound concentrations in ex vivo islets, the effect was pronounced on fasting insulin levels in NHPs following acute dosing. Interestingly, these animals were not hypoglycemic leading to our hypothesis of the counterbalancing glycemic effects of FFAR1-induced glucagon secretion. A second component of the FFAR1 mechanism we observed in our studies is enhanced secretion of GLP1, which may also contribute to the observed weight loss. This leads to the potential of a combined secondary effect of FFAR1 agonism – weight loss through enhanced GLP1 and glucagon secretion (34). This is supported by human data which show increased glucagon and GLP1 secretion with site-3 agonist SCO-267 (22).

While Takeda’s clinical setbacks have delayed and discouraged the pursuit of FFAR1 agonists, the unique differential pharmacology seen with full agonists offers a potential new path forward. Indeed, full agonists are now beginning to reach humans and have already differentiated themselves from partial agonists like TAK-875 both in terms of efficacy as well as safety. The combinability of this mechanism with other existing therapies only amplifies the appeal. Several questions around the mechanism remain to be answered, including how FFAR1 full agonists can sustain such significant levels of insulin secretion. To resolve this, longer term studies in humans will need show continued efficacy over time. While the durability of these compounds have been shown preclinically *in vivo*, even in the face of secondary failure from other common diabetic medications, maintenance of pancreatic insulin content following chronic treatment with compound A in humans should be demonstrated. Nevertheless, the remarkable ability of this class to restore insulin secretion from T2D islets and reduce glucose excursion in diabetic non-human primates serves as a rationale to those willing to undertake the effort to address these questions. GPR40 full agonists have demonstrated superior pharmacology to partial agonists with a differentiated and robust therapeutic effect on islet (insulin and glucagon) and gut hormone secretion (GLP1). Recently, GLP-1 receptor agonists, have been confirmed to reduce all-cause mortality and worsening kidney function in patients with type 2 diabetes and ongoing clinical trials have shown promising results in patients with non-alcoholic steatohepatitis (NASH), chronic kidney disease (CKD) and are being explored in Alzheimer’s disease suggesting additional potential indications besides T2D and obesity for mechanisms that potentiate activation of GLP1 receptor (35–38). Future clinical studies are warranted to establish GPR40 full agonists as a novel and safe therapeutic approach for the treatment of these diseases.

## Data availability statement

The raw data supporting the conclusions of this article will be made available by the authors, without undue reservation.

## Ethics statement

The animal study was reviewed and approved by IACUC Janssen.

## Author contributions

BR designed and conducted experiments, analyzed data, and wrote manuscript. JL, HH, IB, and JQ designed and conducted experiments and advised on manuscript. PL designed experiments and analyzed data. TM designed and conducted experiments and analyzed data. LN designed experiments and analyzed data. MP analyzed data and advised on manuscript. AP analyzed data and advised on manuscript. All authors contributed to the article andapproved the submitted version.

## Acknowledgments

The authors would like to thank Jose Oberholzer and Yong Wang formerly of the University of Illinois at Chicago currently of the University of Virginia for supplying human islets and technical expertise.

## Conflict of interest

All authors are employees of Johnson & Johnson – Janssen R&D LLC.

## Publisher’s note

All claims expressed in this article are solely those of the authors and do not necessarily represent those of their affiliated organizations, or those of the publisher, the editors and the reviewers. Any product that may be evaluated in this article, or claim that may be made by its manufacturer, is not guaranteed or endorsed by the publisher.
